# Single replica spin-glass phase detection using field variation and machine learning

**DOI:** 10.1371/journal.pone.0335503

**Published:** 2025-12-03

**Authors:** Ali Talebi, Mahsa Bagherikalhor, Behrouz Askari, G. Reza Jafari

**Affiliations:** Department of Physics, Shahid Beheshti University, Evin, Tehran, Iran; Purdue University, UNITED STATES OF AMERICA

## Abstract

The Sherrington-Kirkpatrick (SK) spin-glass model exhibits well-studied phase transitions that are mostly established using replica-based methods. Regardless of the method used for detection, the intrinsic phase of a system exists whether or not replicas are considered. Therefore, in this study, we propose a novel method for phase detection based on the variation of the local field experienced by each spin in a configuration of a single replica. The mean and the variance of these local fields are powerful indicators that effectively distinguish different phases, including ferromagnetic, paramagnetic, and spin-glass phases. By analyzing the mean and variance of these local fields, we develop a machine learning algorithm to generate the phase diagram, which shows strong agreement with the theoretical solutions for the SK model. This algorithm offers a more computationally efficient approach for phase detection in spin-glass systems.

## Introduction

Spin-glasses are disordered magnetic systems with both ferromagnetic and antiferromagnetic coupling between pairs of spins, leading to frustration in updating spins to reach a stable state. Studying these complex systems has long been a challenge for researchers, yet their investigation has led to significant advancements across diverse scientific disciplines, including condensed matter physics [1–3], materials science [4,5], neuroscience [6,7], quantum computing [8–14], and higher-order interactions [15–18] with application in information theory [19]. The first model to describe the behavior of spin-glass systems was the Edwards-Anderson (EA) model [20]. Its formulation includes the interaction of a spin with its nearest neighbors. Later, Sherrington and Kirkpatrick proposed a mean-field simplification of the EA model with long-range interactions, which was exactly solvable [21,22].

Researchers have developed various methods to understand complex systems better. Some of them, to deal with the intricacies of spin-glass systems, are the Replica symmetry method (RS) [23–25], Replica Symmetry Breaking (RSB) [26,27], TAP [28], and the Cavity method [29]. In the RS method, the free energy calculation is simplified by considering multiple replicas of the system and averaging over them, assuming that the replicas are independent and identical. While this trick works well at high temperatures, it faces errors in low temperatures, and fails to predict the nature of the spin-glass phases [19,26,30]. In 1979, Parisi introducedthe RSB solution to overcome the limitations of the RS method [24,26,31–33]. Replicas are no longer identical, providing a more accurate description of the spin-glass behavior. This method can successfully reveal many well-separated local minima of the system, corresponding to different systems’ configurations, and plays a crucial role in understanding the phases of the SK model. The quantities used to identify different phases of the system are called order parameters. In spin-glass systems, magnetization (*m*) and overlap (*q*) determine the system’s phase, where magnetization defines a measure of the average magnetic moment per spin, and the overlap measures the similarity between two replicas of the system.

The mean-field approach to study spin-glass systems was introduced by Thouless, Anderson, and Palmer [28], leading to the TAP equation. The cavity mthod, was developed by Mézard, Parisi, and Virasoro, in which the average field applied to one spin of the network from other spins of the network was related to the average field applied to the same spin if it was removed from the network [29].

All these approaches are used to unravel the intricate behavior of spin-glass systems and to help us understand their distinct phases. Based on the literature, we identify three key parameters: temperature, mean, and variance of the Gaussian distribution function from which the random interactions *J*_*ij*_ are drawn. However, for phase classification, only two ratios, the mean to standard deviation (std), *J*_0_/*J*, and the temperature to std, *T*/*J*, are the essential independent variables. Detecting different phases of spin-glass systems can be done in various approaches, such as theoretical [28,30,31], numerical [34], experimental [27], or a combination of these methods. In this study, we use a numerical approthathich relies on Monte Carlo simulations of spin configurations.

**Fig 1:**
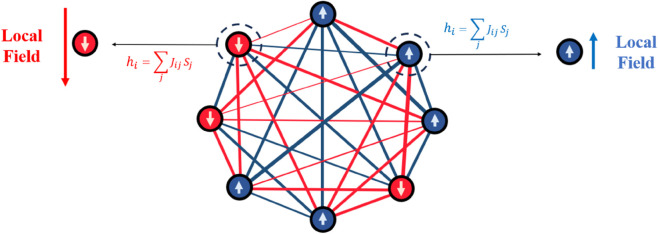
The image shows a system’s configuration, with blue circles representing  + 1 spins and red circles symbolizing –1 spins. Positive and negative interactions are represented by blue and red lines, respectively. Arrows indicate the local fields (in a pre-equilibrium state) calculated from Eq 3, with their lengths illustrating the field’s magnitude and their directions showing its orientation.

According to Parisi’s RSB solution, the assumption that replicas are not identical allowed him to reconstruct the phase diagram of the SK model. In contrast, while multiple replicas are typically needed to explore phases, our study focuses on a single replica with quenched random couplings. Specifically, we examine the local field felt by each spin due to interactions with all other spins in a snapshot of the system (Fig 1). In spin-glass systems, the strong disorder in the couplings leads to significant variations in these local fields from spin to spin. We demonstrate that both the mean and variance of the local field measured here from a single configuration before equilibrium play a pivotal role in distinguishing between spin-glass, ferromagnetic, and paramagnetic phases. Furthermore, we develop a machine learning algorithm that facilitate phase detection,based on the similarity measurement.

In addition, the SK model features a mixed phase in which both ferromagnetic and spin-glass orderings coexist. The boundary of this phase, referred to as the AT-line by Almeida and Thouless [35], specifies the transition between the spin-glass and ferromagnetic phases, marking the onset of the replica symmetry breaking and the presence of a glassy state. The area between the AT-line and the spin-glass phase is identified as the mixed phase, characterized by the instability of the solutions derived from the RS method of the SK model. Subsequently, Parisi [24,31–33] was able to establish a distinct boundary between the spin-glass phase and the mixed phase by exploring the concept of the RSB. For this purpose, we introduce a similarity measure that quantifies how closely each configuration is similar to other well-known phases, which enables us to identify which region of the mixed phase is more closely related to the spin-glass, ferromagnetic, or paramagnetic phases.

### SK model

In this section, we review the approaches proposed by researchers to explore the complexities of spin-glass and to deepen our understanding of their phase transitions. Let’s consider the Hamiltonian of the SK model in the absence of an external field,

$$H(J_{ij},\{S_{i}\})=-\sum_{i<j}J_{ij}\;S_{i}\;S_{j},$$ (1)

where *S*_*i*_ and *S*_*j*_ are Ising spins taking the values {±1} and *J*_*ij*_ is the interaction between distinct pair of spins (*i*,*j*) with i≠j, and comes from a Gaussian Probability distribution with mean *J*_0_/*N* and variance *J*^2^/*N* ensuring that the Hamiltonian is an extensive quantity. Depending on the ratio of mean to std of this distribution, *J*_0_/*J*, and the ratio of temperature to std, *T*/*J*, the system is placed in one of paramagnetic, ferromagnetic, spin-glass, and mixed phases.

Phase transitions in the spin-glass system described by the Hamiltonian in Eq (1) are observed when changes in temperature or in the ratio of the mean to std of the couplings cause the system to shift from one magnetic phase to another; transitioning from a paramagnetic state to a spin-glass phase determined by ratio of thermal fluctuation to std *T*/*J* and from the spin-glass to the ferromagnetic phase, determined by the ratio *J*_0_/*J*.

The first effort to study the phase diagram of the SK model was based on the replica symmetric approach. This method simplifies the calculation of the expected value of the logarithmic partition function into a more manageable calculation of the expected value of the partition function to the power of *n*, where *n* is an integer and number of replicas of the system. Its solution typically includes phases characterized by different magnetic orders and transitions between them. The replica method solution results in two equations of state for the ferromagnetic order parameter, $m=\langle S_{i}^{\alpha }\rangle$, which is an indicator of the alignment of spins and the spin-glass order-parameter, $q=\langle S_{i}^{\alpha }S_{i}^{\beta }\rangle$, in which $S_{i}^{\alpha }$ and $S_{i}^{\beta }$ are the spin states at site *i* in two different replicas *α* and *β* and due to replica symmetric assumption they reduce to $m=\langle S_{i}\rangle$, $q=\langle S_{i}S_{i}\rangle$. The overlap in the simulation approach is treated as a dynamical parameter defined as $q=\lim_{t\to \infty }\lim_{N\to \infty }\left[\left\langle S_{i}\left(t_{0}\right)S_{i}\left(t_{0}\;+\;t\right)\right\rangle \right]$ [19], which shows multivalley structure in free energy of this system, and when the system finds the global minimum of the free energy, it becomes frozen, and the value of this parameter reaches  + 1.

By failure of the assumption of replica symmetry at low temperatures, the RSB method considers different values for *m* and *q* depending on replica indices *α* and *β* as $m_{\alpha }$ and $q_{\alpha \beta }$ to prevent the unphysical conclusion of the replica symmetric solution. Therefore, *q* is not a single value but a distribution in the RSB approach and is a critical order parameter for detecting the spin-glass phase.

### TAP and cavity method

In the TAP equation [28] the average field felt by each spin depends on the magnetization of other spins in the network, which is the ensemble average of the sign of a spin in the network. This method is based on an effective field, $h_{i}=\sum_{j\neq i}J_{ij}\;\langle S_{j}\rangle$, experienced by each spin caused by its neighbors, which is known as the mean-field theorem. The study of the TAP variational principle concerning the SK model is a mean-field technique that focuses on the following self-consistent equation

$$m_{i}=\tanh\beta \left(\sum_{j}J_{ij}m_{j}-\beta \sum_{j}J_{ij}^{2}\left(1-m_{j}^{2}\right)m_{i}\right),$$ (2)

which is a theoretical approach to identify the different phases without replicas. This method is limited to the Ginzburg criterion valid for dimensions higher than 4, and is suitable for pairwise interactions. In the cavity method [29], the local field is used for finding a relation between the effective field and the effective cavity field to regenerate the TAP self-consistent equation. Overall, these existing methods rely on ensemble averaging to evaluate local fields in spin-glass systems. In the following section, we address our proposed local field, which is based on a single configuration of the system.

## Methods

### Single replica method

While the aforementioned approaches address the complexity of the spin-glass phase and rely on replicas, either through RS or RSB, the intrinsic properties of a system exist independently of the detection method. Traditional methods analyze phase transitions using conventional order parameters such as magnetization and overlap. In contrast, we investigate the system’s behavior using only a configuration of a single replica by defining a local field that can reconstruct the phase diagram in close agreement with theoretical predictions. This approach enables us to examine the system’s configuration before equilibrium to determine its phase. In our method, the field each spin experiences differs from that of the others due to the random interactions represented by *J*_*ij*_. The variation in the experienced field by each spin plays a vital role. We classify different phases by the mean and the variance of the local field each spin feels in disordered systems. Our results show that these quantities serve as excellent indicators for accurately identifying the various phases of the system. According to Hamiltonian 1, each spin experiences a local field due to its interactions with others. The field that is applied to the spin *S*_*i*_ by the other spins can be written as

$$h_{i}=\sum_{j\neq i}J_{ij}\;S_{j},$$ (3)

where the summation over $J_{ij}S_{j}$ yields different values for each spin, as the interactions are random values that are drawn from a Gaussian probability distribution. We used a local field, which is calculated in a single configuration of the system, capturing a ’snapshot’ of the system at a state before equilibrium, without adopting a dynamical approach, and it may be better to recognize this method as a morphological method. The mean and the variance of this local field, experienced by each spin in the system with size *N*, can be calculated as

$$\{\langle h\rangle =\frac{\sum_{i\in N}h_{i}}{N},\;Var(h)=\frac{\sum_{i\in N}h_{i}^{2}}{N}-{\left(\frac{\sum_{i\in N}h_{i}}{N}\right)}^{2}.$$ (4)

We utilize the mean and the variance of the local field to develop a machine-learning algorithm that can accurately identify the phase of the system. Our results in the next section highlight the remarkable effectiveness of these two quantities.

## Results

### Simulation

In this section, we will explain the inspiration behind how we identify different phases of the system. We know that the spin-glass model can exhibit three fundamental phases: paramagnetic, ferromagnetic, and spin-glass. We simulate a set of systems in these three distinct regions in extreme limits of ratios, *J*_0_/*J* and *T*/*J*, each region corresponding to one of these phases. In this case, we can track the characteristic behaviors of the system associated with each phase. This observation enables us to distinguish between the three phases and gives us an insight into how the mean and the variance of the local field change from one phase to another.

**Fig 2:**
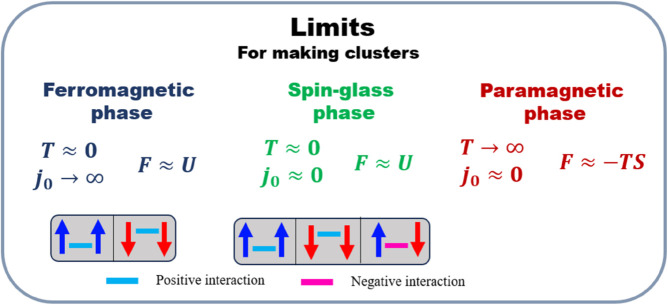
Figure illustrates three extreme limits where paramagnetic, ferromagnetic, and spin-glass phases are expected. Different types of spin-spin interactions that reduce the system’s energy and are more probable to be found inside the system are presented.

By considering the SK model, we simulate a fully connected pairwise interacting system of spins with quenched random couplings and setting *J* = 1. We use the Markov chain Monte Carlo method (MCMC) to update the spins of the system at each temperature. There is no exact threshold for the number of Monte Carlo steps required for a system to reach an equilibrium state. However, we simulate the system for $\lceil \sqrt{(}N^{3})\rceil$ steps to ensure it moves far from the initial configuration. We then simulate the systems across different temperatures and values of *J*_0_ and let the spins update, according to the Boltzmann factor, then we calculated the mean and variance of the local fields for each system using Eq 4. These calculations provide the basis for demonstrating how our algorithm can accurately cluster the systems according to these two quantities. All these simulations and clustering of systems have been carried out based on the information we already had from thermodynamics, and we knew that the system has three phases, and our insights into the different phases stem from our understanding of the Ising model Fig 2.

**Fig 3:**
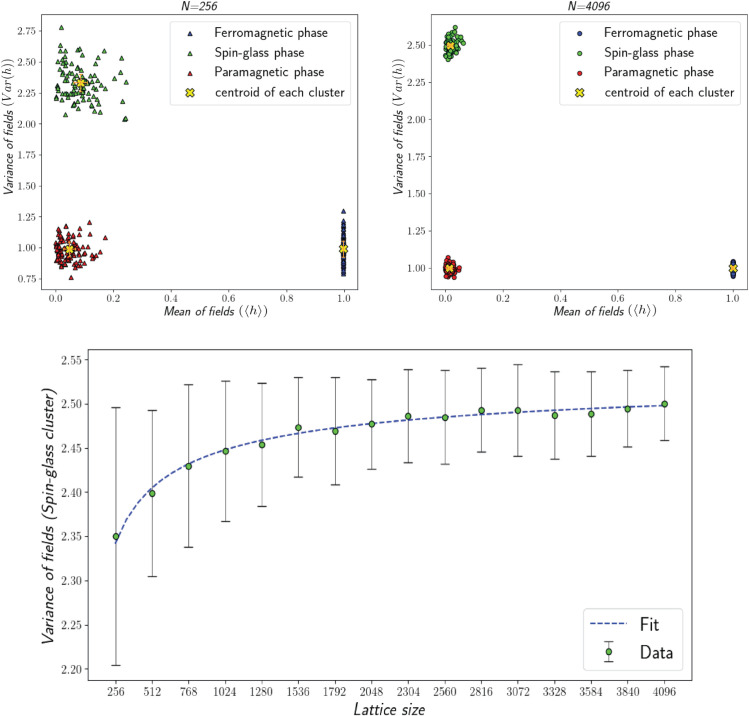
Variance of local fields versus mean of local fields for (a) N=256, (b) N=4096. The centroid of each cluster is marked by an **‘X’** and associated error bars estimated from the standard deviation of point-to-centroid distances. Red, green, and blue clusters represent the paramagnetic, spin-glass, and ferromagnetic phases, respectively. The standard deviation of each cluster centroid is represented by error bars in both *x* and *y* directions. (c): Variance of the local fields in the spin-glass phase ($J_{0}\approx 0$, T≈0) as a function of lattice size. The dashed line represents the function $y=ax^{b}\;+\;c$ fitted to data.

When the mean value of interactions, *J*_0_, is equal to zero, the Gaussian distribution of interactions exhibits symmetry. By increasing *J*_0_, the count of positive values rises; in the case of a significant increase, all interactions will become positive. This behavior is similar to the Ising model, where we know that at low temperatures, the system transitions into the ferromagnetic phase to minimize the energy. Therefore, a large positive value of *J*_0_ in conjunction with low temperatures suggests the emergence of the ferromagnetic phase. To simulate a system in this region, we construct a network of *N* spins and update the spins of the system at low temperatures, ideally close to zero, while *J*_0_ is a significantly large positive value. The ferromagnetic cluster phase is simulated within the range $J_{0}\in [1000,1000.01)$, and the temperature is close to zero T∈(0,0.01). After $\lceil \sqrt{(}N^{3})\rceil$ steps, we calculated the mean and variance of local fields. For each considered range of *J*_0_ and *T*, we simulated 100 systems to clearly demonstrate each cluster, so that each cluster consists of 100 points corresponding to independent systems, Fig 3a, 3b. Note that in the ferromagnetic phase, the value of *J*_0_ is very large so the *J*_*ij*_s are large positive values. As a result, according to Eq 4, the mean value of the local field also becomes large. Since different samples within the ferromagnetic cluster are of the same order, we rescale the calculated mean local field by *J*_0_ to allow meaningful comparison across samples.

By raising the temperature, the system seeks a configuration that maximizes entropy, favoring the configuration with the highest entropy. In this context, the system exhibits a paramagnetic phase. In this case, we simulate a network of spins at high temperatures while *J*_0_ is close to zero. The limited region that we are simulating the extreme limit of paramagnetic systems is done within the range $J_{0}\in (0,0.01)$ and T∈[1000,1000.01). After $\lceil \sqrt{(}N^{3})\rceil$ updates, we calculated the mean and variance of the local fields. The resulting paramagnetic cluster shown in Fig 3a, 3b was obtained by simulating 100 systems in the considered range for *J*_0_ and *T*.

When $J_{0}\approx 0$ and the temperature is low, a new phase known as spin-glass emerges. In this phase, the system tends to display the configuration in which opposite spins connect through negative interactions, while spins that align in the same direction tend to be connected via positive interactions. Consequently, a partial bipolar configuration can be established. It is not a complete bipolar configuration, such as a model in which interaction can be updated [36]. The effect of this partial bipolar configuration is reflected in the variance of the local fields. To probe this phase, we simulate a group of systems running within the range $J_{0}\in (0,0.01)$ and T∈(0,0.01). We update the spins of the system at low temperatures, ideally close to zero, while *J*_0_ is also close to zero. After $\lceil \sqrt{(}N^{3})\rceil$ updates, we calculated the mean and variance of the local fields experienced by each spin, after doing a simulation for 100 systems, the spin-glass cluster appears, as shown in Fig 3a, 3b. After simulating the system in three distinct limits, we constructed the feature space where the x-axis represents the mean and the y-axis represents the variance of local fields. Then we applied the K-means algorithm to find the centroid of each cluster related to different phases in this space. To quantify the statistical robustness of the clustering, we calculated the spread of points within each cluster relative to their centroid. For each cluster, the standard deviation of the point-to-centroid distances was computed along both the *x*-and *y*-directions and represented as error bars on the centroids in Fig 3a, 3b. The corresponding values are reported in Table 1, where the standard deviations are expressed as pairs $\left(X\;\pm \;\sigma_{x},\;Y\;\pm \;\sigma_{y}\right)$, where *X* and *Y* denote the centroid coordinates, and $\sigma_{x}$ and $\sigma_{y}$ represent the standard deviations along the *x*- and *y*-directions, respectively. As expected, these error bars decrease with increasing system size, further confirming the stability and accuracy of our clustering approach.

**Table 1:** The centroid position with associated standard deviations for each cluster at different system sizes. Values are expressed as $\left(X\;\pm \;\sigma_{x},\;Y\;\pm \;\sigma_{y}\right)$, where *X* and *Y* denote the centroid coordinates, and $\sigma_{x}$ and $\sigma_{y}$ represent the standard deviations along the *x*- and *y*-directions, respectively. Smaller error bars at larger *N* indicate improved stability and clustering accuracy.

Centroid of each cluster with error bars ($X\;\pm \;\sigma_{x},\;Y\;\pm \;\sigma_{y}$)		
	**(N = 256)**	**(N = 4096)**
Spin-glass cluster	(0.079 ± 0.068, 2.336 ± 0.154)	(0.021 ± 0.015, 2.494 ± 0.038)
Paramagnetic cluster	(0.055 ± 0.037, 0.990 ± 0.083)	(0.011 ± 0.009, 0.998 ± 0.023)
Ferromagnetic cluster	(0.996 ± 0.000, 1.001 ± 0.080)	(1.000 ± 0.000, 1.000 ± 0.020)

In Fig 3c, the variance of the fields in the spin-glass phase is plotted according to the lattice size to examine the size effect on the centroid and scattering of the data. To estimate the convergence of the variance of the local field in this phase, we used two mathematical models: an exponential function $y=a^{\prime }e^{b^{\prime }x}\;+\;c^{\prime }$, and a power function $y=ax^{b}\;+\;c$. The fitted parameters were $a^{\prime }=-0.186$, $b^{\prime }=-0.001$, and $c^{\prime }=2.496$ for the exponential model and *a* = −3.957, *b* = −0.538, *c* = 2.543 for power model. Both models suggest that the centroid tends to converge towards a constant value for large lattices, with the exponential function indicating a convergence of 2.496±0.358 and the power model predicting 2.543±0.084. Because of its smaller error, the power-law model provides a more accurate description of the convergence. Moreover, since the distance of each point to the centroid of each cluster is measured relatively, the size effect can only improve the clustering accuracy.

**Fig 4:**
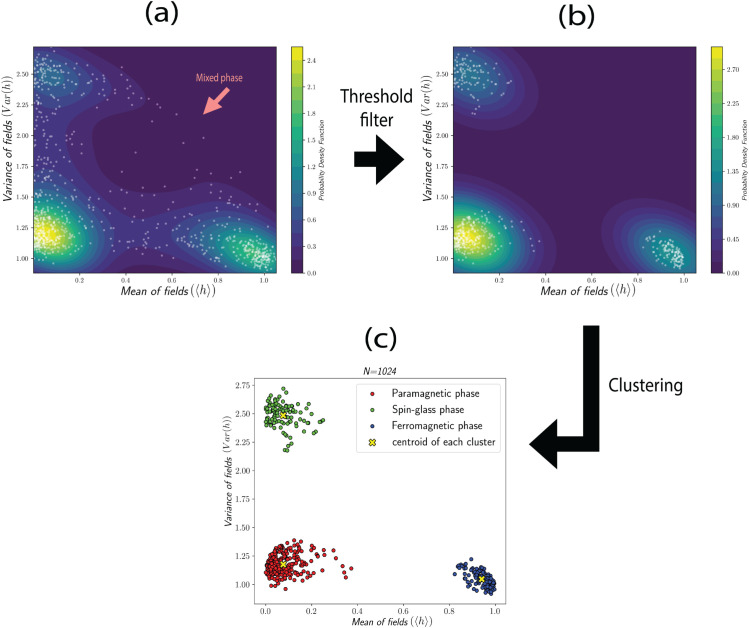
(a): These systems were simulated for $J_{0}/J\in$ (0,3] and T/J∈ (0,3] without the prior knowledge about different phases, and the probability density function of points in feature space illustrates the dense areas as lighter color with a contour-plot. The points stretch from the dense region corresponding to the spin glass phase (left top) to the dense region corresponding to the ferromagnetic phase (right bottom), representing the mixed phase region. (b): The dense areas are separated by removing the data less than threshold = 0.5 in the probability density function. (c): The centroid of each cluster is determined by the K-means algorithm.

Notice that in the first step of our method, we relied on prior knowledge of thermodynamics to simulate the system in different extreme limits Fig 2, where we expect that our two defined quantities, the mean and the variance of the local field, capture three phases. However, if determining these limits is not convenient based on the physical intuition, there are two alternative methods for identifying the clusters in feature space without prior knowledge of thermodynamics. For the first alternative method, we need to randomly select a temperature and the parameters directly used in the Hamiltonian, which in the SK model are *J*_0_ and *J*, then make a simulation by using the MCMC algorithm for $\lceil \sqrt{(}N^{3})\rceil$ steps to track the behavior of the system in a pre-equilibrium state. By repeating this procedure, we find that the feature space is naturally separated into distinct areas. These dense areas in the feature space correspond to three phases of ferromagnetism, paramagnetism, and spin-glass. To make these areas explicit, we compute the probability density function using the kernel estimation method to identify these high-density areas by removing the data with values below a certain threshold. Finally, we apply the K-means algorithm to the remaining high-density data, producing the clustering shown in Fig 4.

**Fig 5:**
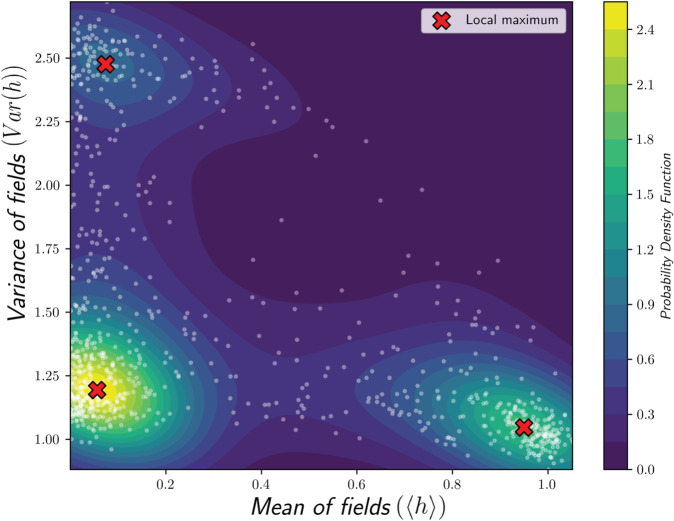
The probability density function of points in feature space of variance versus mean of the local field for $J_{0}/J\in$ (0,3] and T/J∈ (0,3] displayed as a contour-plot. The maxima of the function are marked by an **‘X’**.

The second alternative method for finding clusters is similar to the first, with a difference in the last step. As before, we randomly select a temperature and the Hamiltonian parameters, which in the SK model are *J*_0_ and *J*, and simulate the system using the MCMC algorithm for $\lceil \sqrt{(}N^{3})\rceil$ steps and analyze the behavior of the system in a pre-equilibrium state. After constructing the probability density function of points in feature space, the maxima of these functions indicate the locations of dense areas, which closely correspond to the centroid of clusters identified in previous methods, Fig 5.

After identifying the three well-known phases and simulating groups of systems in these extreme limits, we next simulate systems for different values of *T*, *J*_0_, and calculate the mean and variance of the local fields. We then use the Euclidean measure Eq 5 to compute the distance between each newly simulated system and the centroids of the existing clusters. Using Eq 6, we calculate the similarity of each point to the cluster centroids, where *ε* is a small constant relative to *J* (set to 0.0001 in our simulations). A system closer to a given centroid is considered more similar to that cluster. For clarity, we provide a pseudo-code representation of the algorithm at the end of this section Algorithm 1.

$$\text{ Distance }=\sqrt{{\left(X-X_{c}\right)}^{2}+{\left(Y-Y_{c}\right)}^{2}}$$ (5)

$$\text{ Similarity }=\frac{\epsilon }{\epsilon +\text{ Distance }}$$ (6)

**Fig 6:**
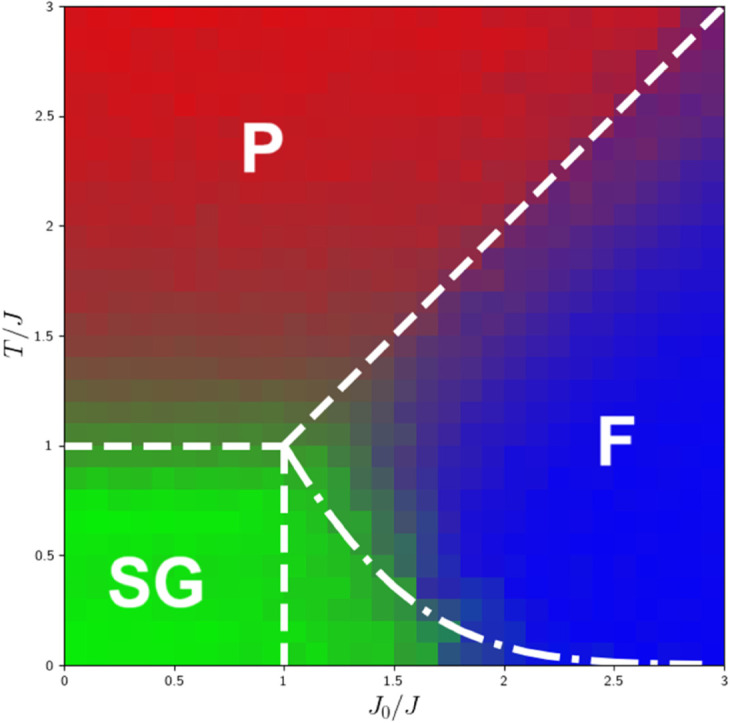
Phase diagram: Dash lines indicate the boundaries of phase transition, obtained from theory, and the dash-dot line marks the AT line. The color of each pixel shows the degree of similarity to one of three phases. Red points indicate similarity to the paramagnetic phase, green points to the spin-glass phase, and blue points to the ferromagnetic phase. The diagram is 30*30 pixels simulated for the lattice size N = 1024.

All these simulations result in the phase diagram Fig 6. The phase diagram is constructed with intervals of *dT* = 0.2 and *dJ* = 0.2, and three colors represent three phases. Note that each pixel is a simulated SK model with specific values of *T* and *J*_0_, and the color of each pixel is determined by the similarity percentage to each of three clusters. To ensure accuracy and remove fluctuations, we repeat the simulation in a loop of 20 samples for each pixel. In Fig 6, the dashed white lines indicate the boundaries of phases which are determined by the exact theoretical solution [21,22,24,31,32], and the dash-dot line shows the AT line [35], the boundary between the mixed phase and the ferromagnetic phase. In this research, we demonstrate that the colored pixels in Fig 6 reproduce the same structure as the theoretical phase diagram.

**Algorithm 1. Single-replica spin-glass phase detection.**



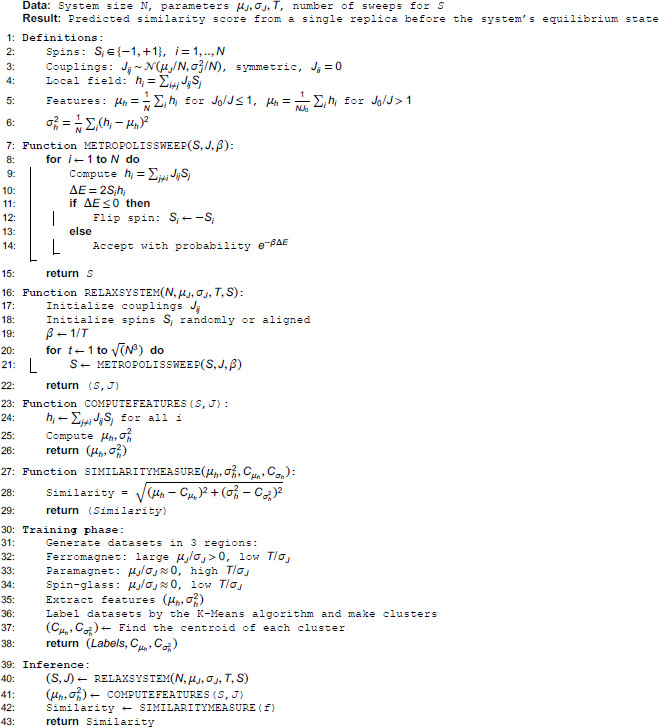



It is worth emphasizing that the standard way to characterize spin-glass phases in the SK model is via the overlap, *q*, so it requires multiple replicas. In addition, determining transition points requires finite-size scaling, and exploring the full phase diagram with traditional Monte Carlo simulations requires reaching equilibration steps, particularly in the spin-glass phase, where equilibration is remarkably slow. Even with simulated annealing or other methods, convergence to the global minimum is not guaranteed. However, our algorithm generates the entire phase diagram with far fewer computational costs. It is based on analyzing the system in a pre-equilibrium state and applying a machine learning framework that distinguishes phases through similarity measures, without requiring replicas or overlap calculations. The close agreement between our predicted phase diagram and the theoretical solution confirms both the efficiency and the accuracy of this approach.

The novelty of our work refers to the single configuration phase detection. This approach allows us to analyze an individual configuration and determine its similarity to three well-known phases. By using the mean and the variance of local fields as two features for clustering, we apply the K-means clustering to identify the centroids corresponding to each phase. Then, we define a parameter that measures the distance of a point from each centroid corresponding to well-known phases and use this parameter as a similarity measure, which is valuable for finding the similarity of the mixed phase to other phases. The region below the AT line where two phases, spin-glass and ferromagnet, coexist is the mixed phase Fig 6. The similarity measurement represents the percentage of similarity of each point in this region to other well-known phases.

## Discussion

Our study focuses on the collective behavior of a well-known complex system, the SK spin-glass model. Due to the disordered interactions between spins, each spin acquires different information through interactions with others. To capture the various phases of this system, our primary goal is to understand the system’s phase without waiting for lengthy iterations of MCMC to reach an equilibrium state. For this purpose, we proposed a local field whose mean and variance serve as excellent indicators for identifying different phases of this system. We trained a machine based on the mean and variance of the local fields to distinguish distinct phases in extreme limits of ratios *J*_0_/*J* and *T*/*J*. Then we simulated SK systems for various values of *J*_0_ and *T* and measured the similarity of each of these systems’ indicators with three well-known phases. According to this approach, we managed to regenerate the phase diagram of the SK model, which, surprisingly, is in great agreement with the well-studied theoretical phase diagram of this system.

The success of our proposed indicators to reproduce the phase diagram efficiently originated in their physical interpretation. In the ferromagnetic phase, spins are strongly constrained to align in the same direction, yielding a larger mean local field compared to the other phases, while the variance remains on the order of *J*. The paramagnetic phase does not restrict spin alignment, and spins are randomly directed, so each spin feels a near-zero mean local field; however, according to the random direction of spins, the variance of the field felt by each spin is close to *J*. For spin-glass, as we know from the phenomenology of the spin-glass, spins feel a partial force of alignment by others that causes them to freeze, producing a near-zero mean local field and a larger variance than either the ferromagnetic or paramagnetic phases. Thus, analyzing the mean and variance of the local fields provides clear insights into the distinct phases of the system.

As displayed in Fig 6, the mixed phase is located below the AT line, with regions near the tail of the AT line exhibiting the behavior of the ferromagnetic phase, while the further areas below the AT line resemble the behavior of the spin glass phase. Clearly, Fig 6 illustrates the phase diagram of the SK model, representing a phase transition from the spin-glass phase to the paramagnetic phase close to *T* = *J*, and a transition from the spin-glass phase to the ferromagnetic phase close to *J*_0_ = *J*. In summary, we should note that all achievements in this study originate from our proposed algorithm, which successfully generates the entire phase diagram of the SK model with much lower computational cost and consequently less required time compared to traditional approaches. The agreement between the generated phase diagram based on our algorithm and the one derived from theoretical solutions confirms the correctness of our method. This success highlights the effectiveness of the introduced local field, which is capable of distinguishing different phases of the system by tracking the behavior of a single configuration, without the need to consider replicas. In addition to the achievements of this approach, there are several open questions for further exploration:

- We have found a new method to detect the phase diagram of the SK model with a machine-learning approach. The probability distribution function of interactions for the SK model is Gaussian. Does this method work for finding the phase diagram of the other spin-glass models with different probability distribution functions of interactions?
- Does the variance of the local field change when the interactions are correlated?
- Can this method be used to determine the phase diagram of the P-spin model?
- Can we use the social, biological, and economic data to predict the phase of these systems, and define an early warning parameter for phase transition?
